# Public health surveillance of Vibrio cholerae in travellers returning to the United Kingdom

**DOI:** 10.1099/jmm.0.002121

**Published:** 2026-02-06

**Authors:** Ching-Ying J. Poh, David R. Greig, Ella V. Rodwell, Claire Jenkins

**Affiliations:** 1 Gastrointestinal Bacterial Reference Unit, UK Health Security Agency, Colindale, London, UK; 2 NIHR Health Protection Research Unit in Gastrointestinal Infections, University of East Anglia, Norwich, UK; 3 Gastro and Food Safety (One Health) Division, UK Health Security Agency, Colindale, London, UK

**Keywords:** cholera, climate change, epidemiology, public health, surveillance, *Vibrio cholerae*

## Abstract

**Introduction.** *Vibrio cholerae* is a diverse species of bacteria that causes watery diarrhoea, vomiting and stomach cramps and is the aetiological agent of cholera.

**Gap statement.** Despite the global upsurge in notifications of cholera and concerns over the impact of climate change, systematic analysis of national and international surveillance data describing the microbiology and epidemiology of *V. cholerae* is sparse.

**Aim.** We reviewed the microbiology and epidemiology of *V. cholerae* isolated from travellers returning to the UK.

**Methodology.** All human isolates of *V. cholerae* detected from 2004 to 2024 were extracted from UK Health Security Agency reference laboratory surveillance systems. Microbiological data were analysed and linked to available epidemiological data and genome sequences for all isolates from 2016 to 2024.

**Results.** There were 984 notifications of *V. cholerae* from 2004 to 2024 (an average of 51 each year), of which 266 (27.0%) belonged to serogroup O1. There were over 180 different sequence types (STs), of which cholera toxin producing ST69 was the predominant type (*n*=99, 28.2%). The highest number of isolates was in 2010 (*n*=74), while the lowest was in 2020 (*n*=8) and 2021 (*n*=4) due to travel restrictions imposed during the COVID-19 pandemic. Children under the age of 10 and the middle-aged and elderly population were most susceptible to infection, and 51.6% of the cases were male. There was a seasonal peak in August and September. Travel was reported by 92.9% of cases, and the most frequently reported travel destinations were India, Pakistan and Kenya.

**Conclusion.** From the UK perspective, to assess the risk to food safety and to more accurately determine the clinical burden of *V. cholerae*, we recommend (i) widespread molecular testing of shellfish to monitor the emergence of *V. cholerae* in UK waters due to climate change and (ii) comprehensive testing of faecal specimens from non-travellers with gastrointestinal symptoms. Public health surveillance and information sharing at the global level is essential to assess the impact of investment in water, sanitation and hygiene initiatives for the prevention of cholera.

## Introduction

*Vibrio cholerae* is a diverse species of bacteria that causes symptoms ranging from mild to watery diarrhoea, vomiting and stomach cramps and is the aetiological agent of cholera. Cholera is an acute gastrointestinal infection where patients pass large volumes of stool that can resemble rice water, leading to severe dehydration that can kill within hours if left untreated [1,2]. Cholera is caused by cholera toxin produced by the prophage-encoded *ctx* genes, which are present in a limited number of *V. cholerae* serogroups, most commonly serogroup O1 [3]. There are over 200 different lipopolysaccharide O antigens, or serogroups, of *V. cholerae *[4]. Although the non-O1 serogroups are associated with a milder form of gastroenteritis, extraintestinal infections can occur, including sepsis, wound infections and otitis media [5,7].

Clinical management of cholera involves oral rehydration therapy, supplemented by treatment with antimicrobials, if required to reduce the severity and duration of the disease [8]. Empirical treatment includes tetracycline, fluoroquinolones, macrolides, co-trimoxazole and, less commonly, extended spectrum beta-lactams [9]. The emergence of antimicrobial resistance in *V. cholerae* has led to increasing global public health concern and poses new challenges in managing cholera treatment [10,11].

Humans become infected with *V. cholerae* via contaminated water, and limited access to clean water and/or sanitation facilities is a driver of transmission. The consequences of a humanitarian crisis, such as the disruption of water and sanitation systems, or the displacement of populations to inadequate and overcrowded camps, can increase the risk of outbreaks. It is estimated that there are 1.3 to 4.0 million cases and 21,000 to 147,000 deaths due to cholera each year [12]. *V. cholerae* has caused seven pandemics of cholera (the seventh is on-going) and remains a significant global threat to public health [13]. Cholera is endemic across Africa and Asia, and outbreaks are common, resulting in a large healthcare burden in these regions [14,15].

The UK Standards for Microbiology Investigations for Gastroenteritis recommends testing faecal specimens for *V. cholerae* in individuals with gastroenteritis reporting recent travel (within 2 to 3 weeks) to countries where cholera is endemic, patients with suspected cholera and/or individuals reporting epidemiological links to outbreaks caused by the consumption of contaminated seafood (Gastroenteritis S7 Syndromic Documents) [16]. Consequently, the true incidence of domestically acquired *V. cholerae* infection in the UK remains unknown, and almost all isolates of enteric origin are associated with travellers' diarrhoea. There is no evidence that the pandemic serogroup *V. cholerae* O1 is endemic in the UK, although non-O1 serogroups have been detected in UK rivers and coastal waters [17].

In 2018, the UK Health Security Agency (UKHSA) implemented whole-genome sequencing (WGS) for the routine surveillance of *V. cholerae* [18]. The aim of this study was to review the microbiology and epidemiology characteristics of *V. cholerae* isolated from travellers returning to the UK.

## Methods

### Epidemiological data

Patient information, including sex, age and recent travel, was collected from laboratory request forms upon submission. These data were stored in the Gastro Data Warehouse, an in-house UKHSA database for storing and linking patient demographic and microbiological typing data. Data on symptoms were limited, stating only that the patient had either gastrointestinal symptoms or an extraintestinal infection. There were no data on the severity of symptoms or patient outcomes. A case was defined as an individual that had *V. cholerae* isolated from their faecal specimen or an extraintestinal site.

### Microbiology

Faecal specimens submitted for microbiological testing from individuals hospitalized and community cases with symptoms of gastrointestinal infection reporting travel within 4 weeks to endemic regions were tested for *V. cholerae*. Positive cultures are referred to the Gastrointestinal Bacteria Reference Unit (GBRU) for confirmation and typing. Prior to 2016, phenotypic identification was performed by inoculating a combination of over 40 biochemical substrates. Utilization of the substrate was identified by a colour change or gas production within the media. The ability (or inability) of the bacteria to utilize the substrates was recorded as a positive (or negative) result and compared with that of published tables categorizing the known reactions of *Vibrio* species. Isolates of *V. cholerae* were agglutinated with antisera raised to O1 (Ogawa and Inaba) and O139 (Bengal) antisera to determine the serogroup.

### Whole-genome sequencing

All viable cultures of *V. cholerae* submitted to the GBRU between January 2016 and December 2024 were sequenced (see Table S1, available in the online version of this article). Genomic DNA was extracted and indexed using Nextera XT DNA and Nextera Flex sample preparation kits (Illumina) and sequenced using the Illumina HiSeq 2500 and NextSeq 1000 platforms at UKHSA. Trimmomatic (v0.36) [19] was used to trim sequence adapters. Reads with a PHRED score below 30 and a read length of less than 50 bp were discarded, along with their paired reads. FASTQ reads from all sequences in this study can be found at the UKHSA Pathogens BioProject at the National Center for Biotechnology Information (BioProject number PRJNA438219).

### Sequence typing and virulence profiling

Sequence type (ST) assignment was performed using MOST v2.18 [20] https://github.com/phe-bioinformatics/MOST. Any multilocus sequence type (MLST) gene sequences that did not match the existing alleles were submitted to pubMLST (see https://pubmlst.org/vcholerae/) for assignment of a new allelic type. Similarly, new allelic profiles were also submitted to the database for assignment of a new ST. GrapeTree was employed to generate a minimum spanning tree (MSTree-V2). This was visualized in the GrapeTree platform and annotated with ST derived from SRST2.

Using GeneFinder v2.9 (https://github.com/ukhsa-collaboration/gene_finder) which utilizes Bowtie2 v2.1 [21] to perform sequence alignment, FASTQ reads were mapped to the virulence regulator gene *toxR* (GenBank accession no. KF498634), the cholera toxin gene *ctxA* (GenBank accession no. AF463401), *wbe*O1 and *wbf*O139 (GenBank accession numbers KC152957 and AB012956) encoding the somatic O antigens O1 and O139, *tcpA* classical and *tcpA* El Tor gene sequences (GenBank accession numbers M33514 and KP187623). The best match to each target was reported with metrics, including coverage, depth and nucleotide similarity in XML format for quality assessment. *toxR* is found in all *V. cholerae* isolates and is regarded as a marker for species identification. The *ctxA* encoding cholera toxin is most often, although not exclusively, associated with *V. cholerae* serotypes O1 and O139 and is a characteristic of these pandemic lineages [22]. Variants of *tcpA* can be used to identify the classical and El Tor biotypes [22]. For *in silico* predictions, only results that matched to a gene determinant at >80% nucleotide identity over >80% target gene length were accepted.

## Results

### Overview of the dataset

**Fig. 1.**
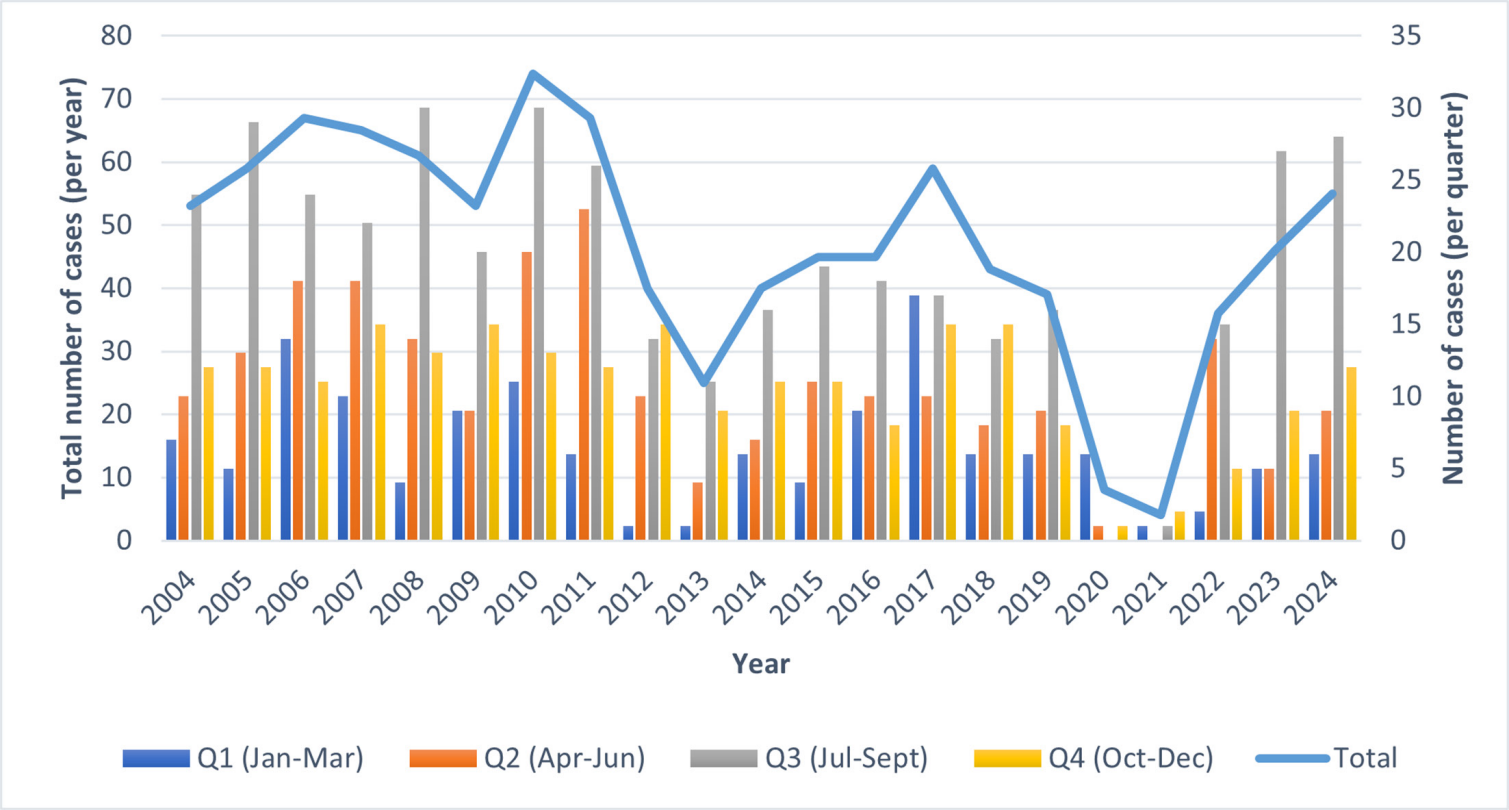
Number of *V. cholerae* cases (organized by quarter) referred to the Gastrointestinal Bacteria Reference Unit between January 2004 and December 2024 (*n*=984). Coloured bars represent different quarters per year. Solid line represents total annual cases.

In total, there were 985 isolates of *V. cholerae* isolated from 984 patients presenting with symptoms of diarrhoeal illness in England submitted to the GBRU between January 2004 and December 2024 (Fig. 1). From 2004 to 2024 (excluding 2020 and 2021), on average, 51 confirmed *V. cholerae* isolates are referred to GBRU for identification and typing each year. The greatest number of isolates was received in 2010 (*n*=74), while the lowest number was received in 2020 (*n*=8) and 2021 (*n*=4) due to travel restrictions imposed because of the COVID-19 pandemic. Of the 12 cases in 2020 and 2021, 9 were reported either before the travel restrictions were imposed in March 2020 or after the restrictions were relaxed in May 2021. The three cases identified during the travel ban were *V. cholerae* non-O1 serogroups; they did not report recent travel outside the UK, and for two of the patients, *V. cholerae* was isolated from extraintestinal sites, including a skin swab and a tracheal aspirate.

**Fig. 2.**
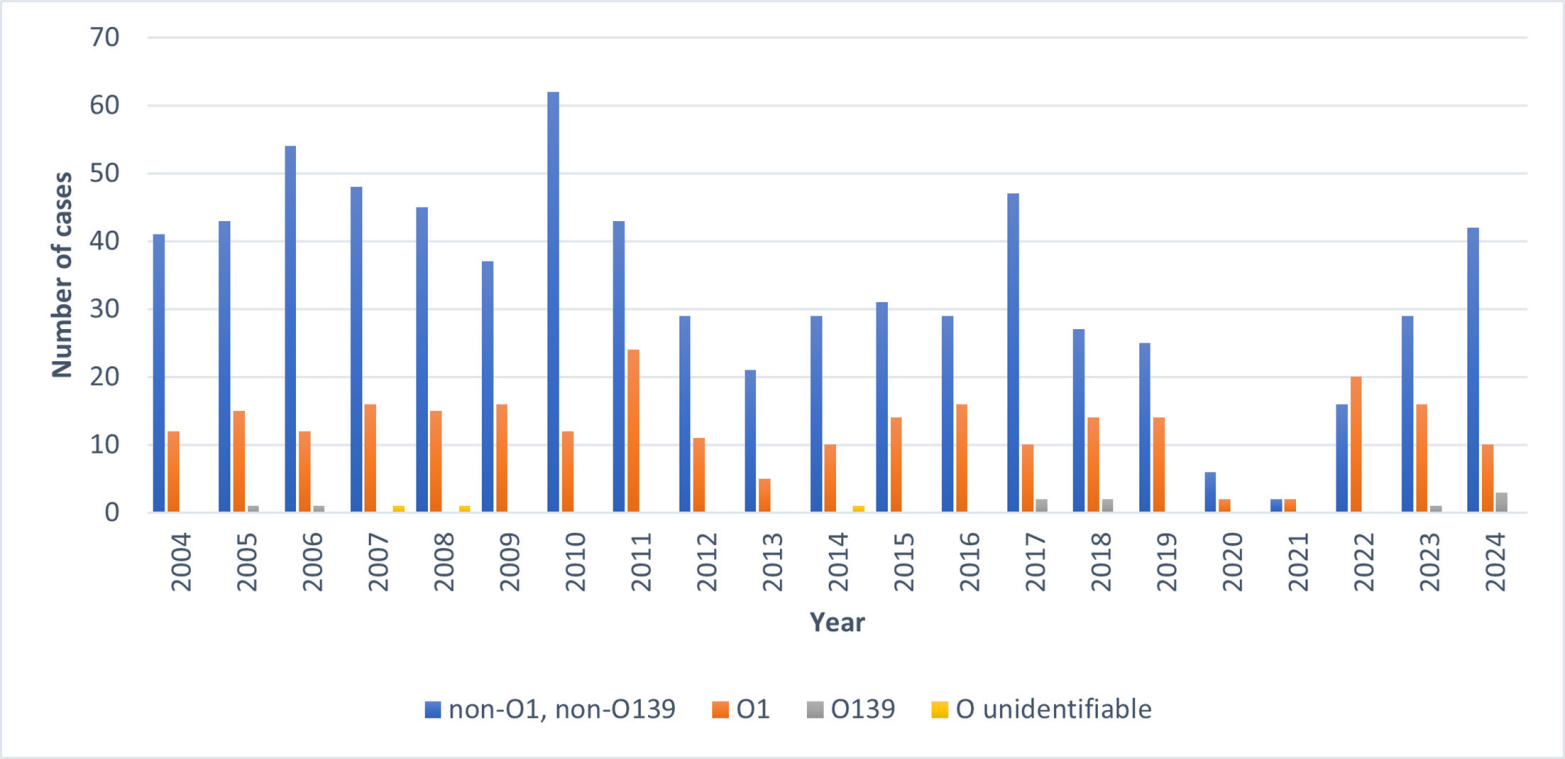
Number of *V. cholerae* cases (categorized by serogroup) referred to the Gastrointestinal Bacteria Reference Unit between January 2004 and December 2024 (*n*=984).

The majority of isolates belonged to the non-O1, non-O139 group (*n*=706, 71.7%), and 266 (27.0%) belonged to the O1 serogroup (Fig. 2). *V. cholerae* O139 was uncommon, with only 10 (1.0%) cases. There were three cases (0.3%) where the serogroup could not be determined and were categorized as ‘O unidentifiable’. For *V. cholerae* O1 cases (*n*=266), the majority had the El Tor biotype (*n*=235, 88.3%), while there were 31 cases (11.7%) with no identified biotype. There were no *V. cholerae* O1 cases with the classical biotype.

### Descriptive epidemiology

**Fig. 3.**
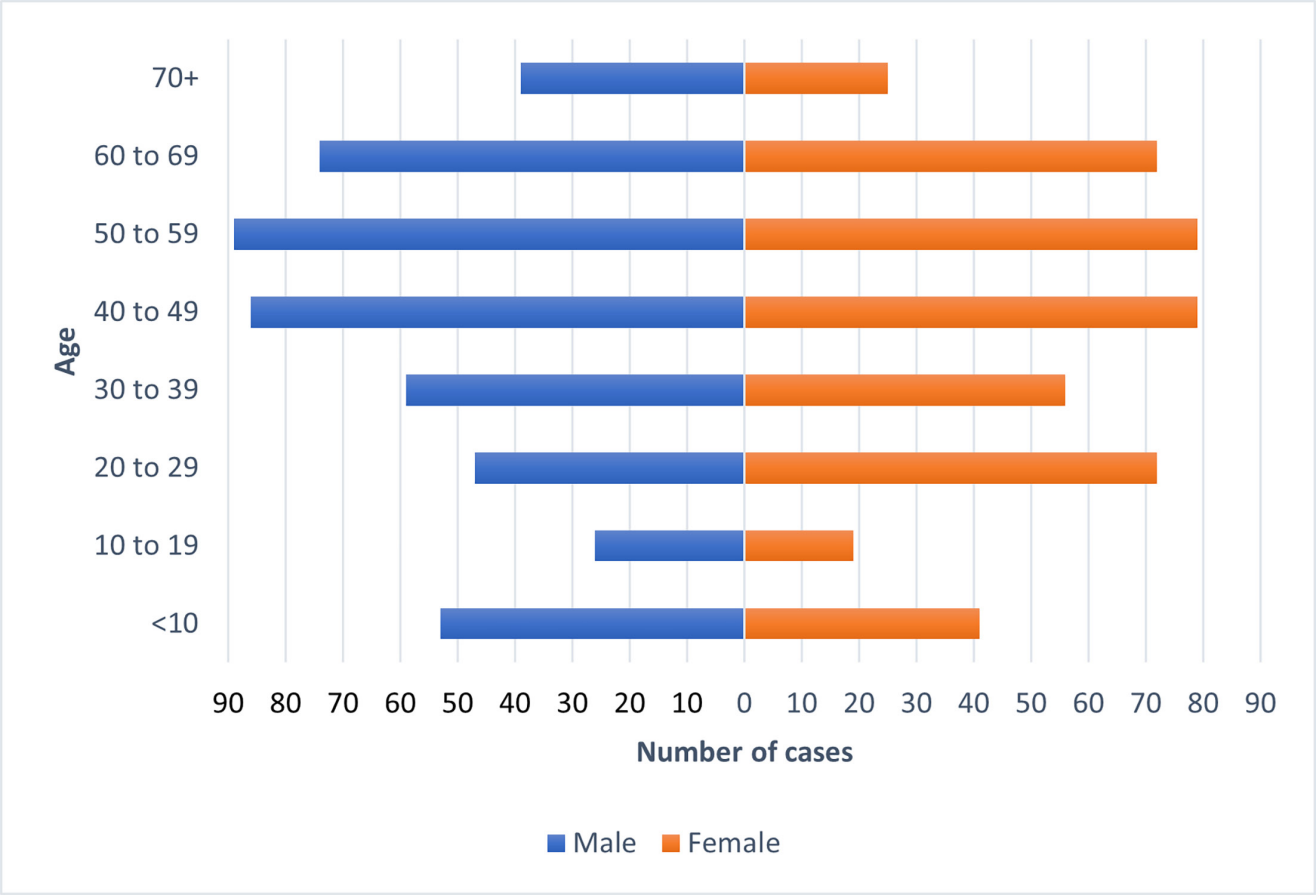
Age–sex distribution of total *V. cholerae* cases reported to UKHSA [isolates originating from England (*n*=916), where date of birth, sample date and sex were available].

The age–sex distribution of all *V. cholerae* cases included in this study where age and sex data were available (*n*=916/984=93%) indicated a balanced sex ratio, although the number of male cases was greater (*n*=473, 51.6%) compared to female (*n*=443, 48.4%). The median age for females was 45 years (IQR: 26–58), while the median age for males was 46 years (IQR: 28–59). Additionally, the highest proportion of cases belonged to the 50 to 59 age group for males and females combined (*n*=168, 18.3%), including for females only (*n*=79, 8.6%) and males only (*n*=89, 9.7%). The second highest proportion of cases was observed in the 40 to 49 age group (*n*=165, 18.0%). The lowest proportion of cases was observed in the 10 to 19 age group for males and females combined (*n*=45, 4.9%), males only (*n*=26, 2.8%) and females only (*n*=19, 2.1%) (Fig. 3).

**Fig. 4.**
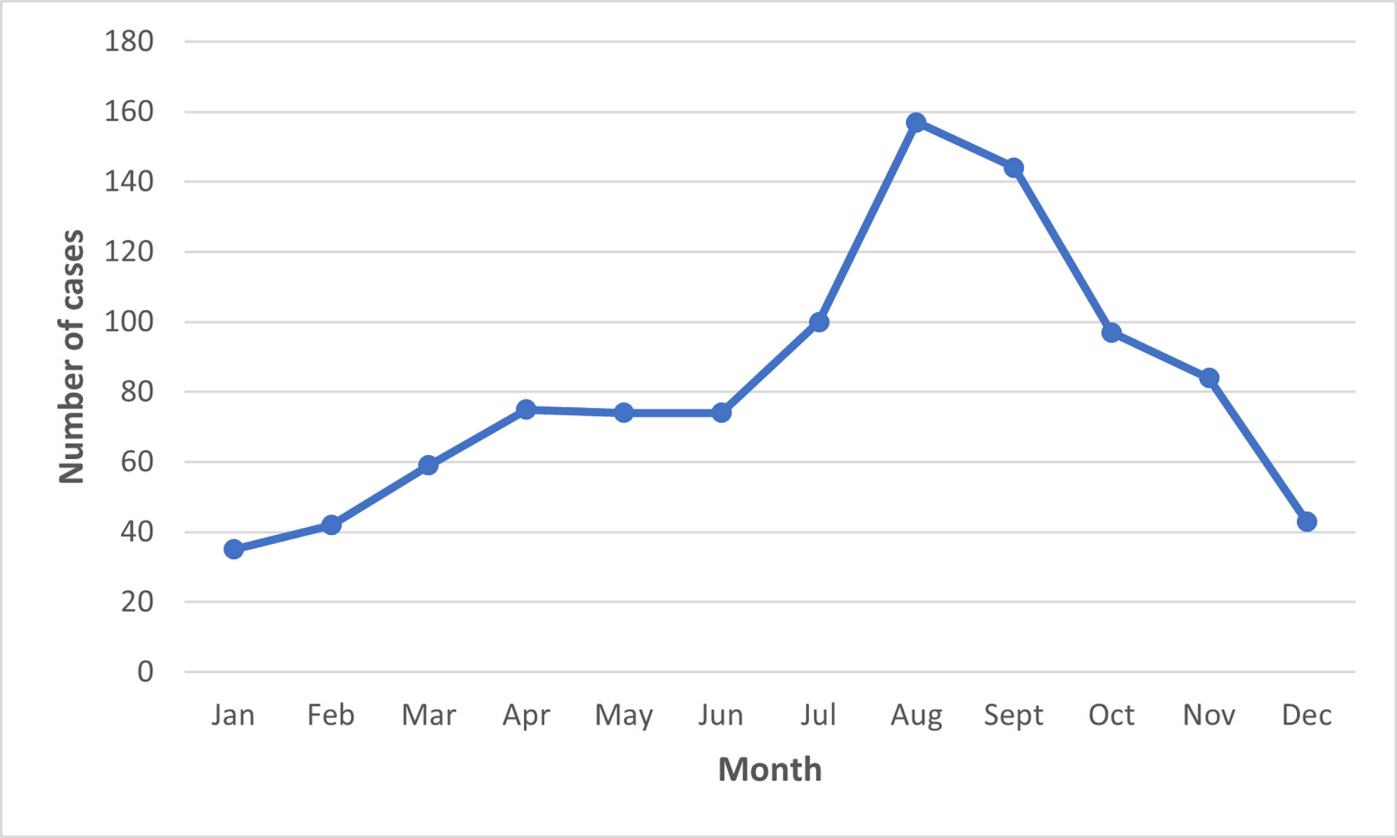
The number of *V. cholerae* cases (*n*=984) by months between 2004 and 2024 in England.

Seasonal variation in the number of reported *V. cholerae* cases was observed, with incidence highest between the summer months of July and September (Fig. 1), forming a late summer peak in August (*n*=157) and lower levels during off-peak seasons from December to February (Fig. 4).

**Fig. 5.**
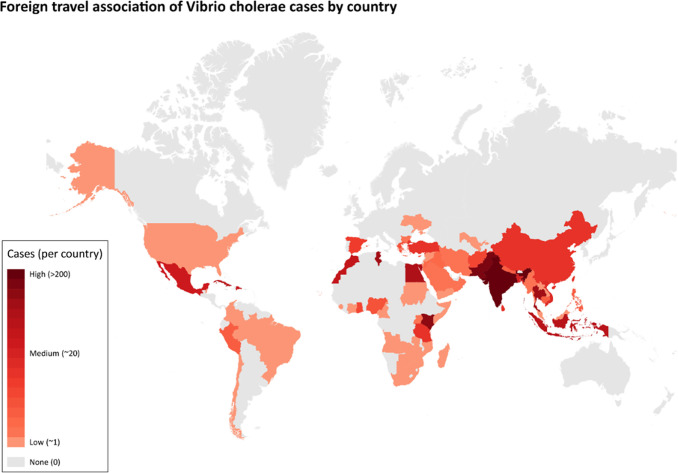
*V. cholerae* cases associated with foreign travel outside of the UK, where a specific country was stated (*n*=868). Countries are colour-coded in proportion to the frequency of cases associated, i.e. the higher the frequency of cases, the darker the colour.

*V. cholerae* cases were detected in all regions of England and one in Wales, with the South of England having the highest frequency (*n*=343, 35.0%) – South East (*n*=220); South West (*n*=123). The North of England had the second highest frequency of cases (*n*=313, 31.9%), specifically the North West UKHSA centre (*n*=144) and Yorkshire and Humber (*n*=111). Excluding the Wales case, the lowest number of reported cases was in the West Midlands (*n*=56, 5.7%) and the North East of England (*n*=58, 5.9%) (Table 1, Fig. 5).

**Table 1.** Geographical distribution of *V. cholerae* cases by UKHSA region and centre (where available) in England and Wales (*n*=980). Asterisk (*) denotes a single case where the patient resided in Wales but was referred to a hospital in England

UKHSA region	UKHSA centre	Cases (%)
London	London	108 (11)
Midlands and East of England	East Midlands	67 (6.8)
	East of England	92 (9.4)
	West Midlands	56 (5.7)
North of England	North East	58 (5.9)
	North West	144 (14.7)
	Yorkshire and Humber	111 (11.3)
South of England	South East	220 (22.4)
	South West	123 (12.6)
Wales*	–	1 (0.1)

The geographical distribution of cases typically clustered around major cities, notably, London, Manchester, Newcastle and Leeds (Fig. 5). One case resided in Wales, while another case resided in the Isle of Man (not shown).

**Fig. 6.**
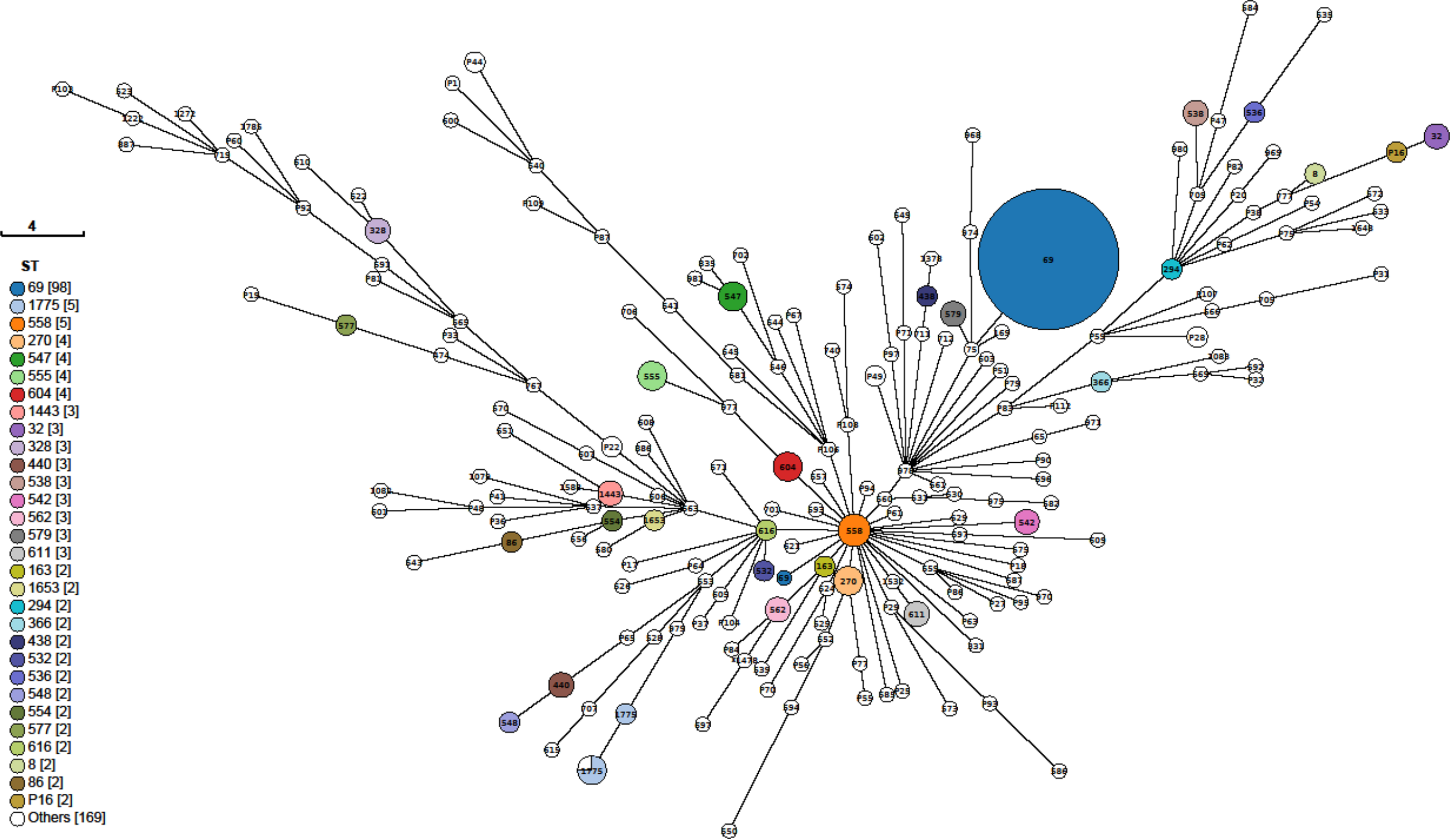
Minimum spanning tree describing the 7-gene MLST of *V. cholerae* samples submitted to UKHSA between 2015 and 2024, where MLST profiles were available (*n*=348). Annotated with labels to describe the ST, and coloured to reflect each ST.

Of the 984 cases, 914 (92.9%) were associated with foreign travel, 14 (1.4%) were not associated with foreign travel, and foreign travel was unknown for 56 cases (5.7%). Overall, foreign travel was most commonly associated with Asia (*n*=564, 57.3%) and Africa (*n*=243, 24.7%) (Table 2). The top three travel destinations were India (*n*=239), Pakistan (*n*=135) and Kenya (*n*=68) (Fig. 6).

**Table 2.** Prevalence of *V. cholerae* cases and their association with foreign travel, categorized by continent (*n*=984)

Continent	No. (%)
Africa	243 (24.7)
Asia	564 (57.3)
Europe	23 (2.3)
North America	62 (6.3)
South America	7 (0.7)
Y – not stated	15 (1.5)
None	14 (1.4)
Unknown	56 (5.7)
**TOTAL**	**984**

### Population structure of *V. cholerae* isolated in the UK from returning travellers

Sequence typing data were available for 351 isolates, of which 229 isolates belonged to the non-O1, non-O139 serogroup, 115 isolates were O1, and 7 were O139. Of these 351 isolates, 66 (18.8%) were novel STs and were assigned either a provisional ST (pST) or were designated as ‘novel ST’. Overall, ST69 was the predominant ST (*n*=99, 28.2%), followed by ST1775 (*n*=7, 2.0%) and ST558 (*n*=5, 1.4%)Fig. 7. There were 180 different STs associated with the non-O1, non-O139 serogroup, while 7 STs were associated with the O139 serogroup (ST163, ST544, ST586, ST707, ST1222, pST93 and pST103). There were 14 different STs associated with the O1 serogroup, of which ST69 was the predominant ST (*n*=98) (Table S1).

Of the 351 isolates with WGS typing data, 104 (29.6%) belonged to CC69. Genome-derived MLST indicated that CC69 isolates were consisted of four STs, with the majority of CC69 isolates belonging to ST69 (*n*=99/104, 95.2%). Additional STs included ST579 (*n*=3/104, 2.9%), ST75 (*n*=1/104, 1.0%) and ST169 (*n*=1/104, 1.0%). Of the CC69 isolates, 43 (41.3%) were associated with foreign travel to Pakistan, 26 (25.0%) to India, and 7 (6.7%) to Bangladesh.

## Discussion

In the UK, the majority of faecal specimens from patients presenting to primary healthcare with gastrointestinal symptoms are examined for *V. cholerae* only if they report recent travel to countries where cholera is endemic (Gastroenteritis S7 Syndromic Documents). The decline in notifications of *V. cholerae* in England that started in 2011 was most likely linked to a reduction in travel to endemic regions, possibly due to political unrest in certain countries [23]. The number of cases increased in 2014 and remained stable until 2019. In 2020, notifications fell sharply, and this decline in notifications was linked to the travel restrictions put in place during the COVID-19 pandemic. Once the travel restrictions were lifted, notifications quickly returned to pre-pandemic levels [24].

Based on global surveillance data from 2022, the World Health Organization reported a global upsurge of cholera caused by *V. cholerae* O1, with more cases reported from an increasing number of countries [10,25]. There were more outbreaks, notably in Afghanistan, Cameroon, the Democratic Republic of Congo, Malawi, Nigeria, Somalia, Syria and Yemen, and the size of the outbreaks also increased [15,26,31]. An upsurge of *V. cholerae* O1 was also captured in European surveillance systems [10], and in the trends analysis in this study, where the number of notifications of *V. cholerae* O1 in 2022 was higher than they had been for over 10 years. This increase may be due in part to the relaxation of travel restrictions providing the opportunity for European nationals to visit family and friends living in endemic regions, following the hiatus caused by the COVID-19 pandemic.

Although, in the UK, the notifications of *V. cholerae* O1 have declined over the last 2 years, the notifications of non-O1 *V. cholerae* – which includes *V. cholerae* O139 and *V. cholerae* non-O1, non-O139 – have continued to increase, with numbers in 2024 at the highest they have been since 2017. Undoubtedly, transmission of *V. cholerae* is linked to the lack of adequate safe water and sanitation due to underdevelopment, poverty and conflict [29]. However, national and international agencies, including the WHO, the European Food Safety Authority (EFSA) and the Food Standards Agency (FSA) in the UK, have all highlighted concerns that climate change may be a contributing factor. There is good evidence that extreme weather events, such as floods, droughts and cyclones, trigger new outbreaks and worsen existing ones [2,32, 33]. Warmer waters are also likely to result in increased *Vibrio* species that accumulate in fish, shellfish and crustaceans [7,34, 35].

*Vibrio* species have been associated with foodborne illness when contaminated shellfish are consumed raw or lightly cooked [36 37, 38, ]. Individuals with a weak immune system are at risk from *V. cholerae* O1 and non-O1 serotypes [39,42]. Although shellfish are not currently routinely screened for *Vibrio* species by the food industry, FSA investigated five UK incidents involving *Vibrio* in shellfish products reported during 2022 and 2023. Four of the shellfish products were imported, while the fifth incident was the first report of *V. cholerae* from shellfish sourced from UK waters since records began [45, 43]. However, there have been no *Vibrio*-related foodborne outbreaks reported in the UK in recent times (UKHSA in-house data).

Analysis of the population structure reveals that *V. cholerae* is a diverse species. The presence of cholera toxin gene *ctxA* is almost exclusively associated with *V. cholerae* O1. A notable exception was the strain of *V. cholerae* serogroup O139 that emerged in the Bay of Bengal region in the early 1990s [44]. However, since that time, notifications of *V. cholerae* O139 have declined globally, and the isolates *V. cholerae* O139 detected in this study were negative for *ctxA*. Although almost all *V. cholerae* O1 *ctxA* isolates belonged to ST69, the non-O1 non-toxin-producing serogroups belonged to a large number of different STs, with very few STs comprising more than one isolate. Within the STs that comprise multiple isolates, the associated cases report travel locations in Africa, Asia, the Americas and Europe, indicating that common STs are widely geographically dispersed. Other studies have described the diversity of travel-associated non-pandemic *V. cholerae* [4,45].

The age–sex distribution analysis in this study indicates that in UK residents, children under the age of ten and the middle-aged group are most susceptible to infection and/or most at risk of experiencing severe clinical outcomes requiring medical care. The peak of case numbers observed in August and September is most likely due to increased travel during the summer holiday period and the impact of warmer climatic conditions in the northern hemisphere. Most notifications were from cases reporting travel to India or Pakistan, reflecting both the high incidence of *V. cholerae* associated with the Indian subcontinent and the proportion of UK nationals travelling to this region each year, compared to other endemic regions.

It is universally acknowledged that the key to prevention of cholera is proper investment in water, sanitation and hygiene (WASH); however, surveillance is necessary to assess the impact of WASH initiatives [29]. Accurate diagnostics and early case detection, integrated into strong surveillance systems, are essential tools for the management and control of cholera epidemics. Local investment in genomic surveillance is crucial to determine the origins of cholera outbreaks, monitor the spread of the disease and inform public health approaches to the control of the disease, particularly with regard to cross-border and international collaboration, to prevent ongoing transmission [29]. The WHO recommends that surveillance of *V. cholerae* should be part of an integrated disease surveillance system that includes feedback at the local level and information sharing at the global level [46]. To support these initiatives, it is UKHSA policy to make genome sequencing data linked to *V. cholerae* surveillance generated in the UK publicly available in real-time [47].

Closer to home, there is an increased risk of domestic acquisition of *V. cholerae*. Due to climate change, UK waters have become progressively warmer over the past 100 years, with average winter temperatures in particular increasing over the past 20 years [48]. We recommend widespread molecular testing of shellfish and more comprehensive testing of faecal specimens from non-travellers with gastrointestinal symptoms to monitor the emergence of *V. cholerae* in UK waters and ensure safety in the food chain.

## Supplementary material

10.1099/jmm.0.002121 Uncited Table S1.

## Abbreviations

EFSA: European Food Safety Authority
FSA: Food Standards Agency
GBRU: Gastrointestinal Bacteria Reference Unit
MLST: multilocus sequence type
pST: provisional sequence type
STs: sequence types
UKHSA: UK Health Security Agency
WASH: water, sanitation and hygiene
WGS: whole-genome sequencing
